# Prediction of in-hospital mortality in patients with post traumatic brain injury using National Trauma Registry and Machine Learning Approach

**DOI:** 10.1186/s13049-020-00738-5

**Published:** 2020-05-27

**Authors:** Ahmad Abujaber, Adam Fadlalla, Diala Gammoh, Husham Abdelrahman, Monira Mollazehi, Ayman El-Menyar

**Affiliations:** 1 Assistant Executive Director of Nursing, Hamad Medical Corporation, Doha, Qatar; 2 College of Business and Economics, Management Information Systems, Qatar University, Doha, Qatar; 3 Industrial Engineering, University of Central Florida, Orlando, USA; 4 Department of Surgery, Trauma Surgery, Hamad Medical Corporation, Doha, Qatar; 5 Department of Surgery, Trauma Surgery, Clinical Research, Hamad Medical Corporation, Doha, Qatar; 6 Department of Clinical Medicine, Weill Cornell Medical College Hamad General Hospital, Doha, Qatar

**Keywords:** Prediction models, Traumatic brain injury, Machine learning approach

## Abstract

**Background:**

The use of machine learning techniques to predict diseases outcomes has grown significantly in the last decade. Several studies prove that the machine learning predictive techniques outperform the classical multivariate techniques. We aimed to build a machine learning predictive model to predict the in-hospital mortality for patients who sustained Traumatic Brain Injury (TBI).

**Methods:**

Adult patients with TBI who were hospitalized in the level 1 trauma center in the period from January 2014 to February 2019 were included in this study. Patients’ demographics, injury characteristics and CT findings were used as predictors. The predictive performance of Artificial Neural Networks (ANN) and Support Vector Machines (SVM) was evaluated in terms of accuracy, Area Under the Curve (AUC), sensitivity, precision, Negative Predictive Value (NPV), specificity and F-score.

**Results:**

A total of 1620 eligible patients were included in the study (1417 survival and 203 non-survivals). Both models achieved accuracy over 91% and AUC over 93%. SVM achieved the optimal performance with accuracy 95.6% and AUC 96%.

**Conclusions:**

for prediction of mortality in patients with TBI, SVM outperformed the well-known classical models that utilized the conventional multivariate analytical techniques.

## Background

Traumatic Brain Injury (TBI) is defined as the brain injury that is caused by external trauma [1]. TBI causes death and disabilities more than any other trauma [2]. The life expectancy reduces significantly with TBI as the mortality rate increases between 30 and 70% [1, 3] compared to other types of injuries. TBI affects millions of people around the world yearly causing a major global burden [3]. Globally, 64–74 million individuals around the world are estimated to sustain TBI every year with the greatest burden of the disease is in Southeast Asian and Western Pacific regions [2]. Mortality is associated with advanced patient age and the severity of TBI. It was found that the 14-day in-hospital mortality post severe TBI reaches up to 24.5% in adults between 16 and 65 years and greater than 40% in patients over 65 years old [4]. There are several published and widely used prognostic/outcome predictive models that demonstrated good predictive and discrimination power. Table 1 shows some of the widely used prognostic models and their performance as measured by the Area Under the Curve (AUC) [5–15].

**Table 1:** examples of popular TBI prognostic models

Model	Applies to	Objective(s)	Variables	Performance
Trauma Injury Severity Score (TRISS)	Trauma patients treated at hospitals with or without TBI [5]	Calculates the probability of survival	Age, revised trauma score (GCS, systolic blood pressure, respiratory rate), trauma type and Injury severity score (ISS) [5]	• Good discrimination power • Not specifically designed for TBI [6] • Prone to poor performance in severe TBI [5] • AUC in previous studies: 89% [5], 90% [7] and 92% (8)
The International Mission for Prognosis and Analysis of Clinical Trials in TBI (IMPACT)	Adult patients (age ≥ 14 years) with TBI and GCS ≤ 12	Predicts the 6-month mortality and unfavorable outcomes [5]	Age, GCS motor scale, pupils reactivity, hypoxia, hypotension, CT results (epidural or subarachnoid hemorrhage), lab values (blood glucose level and hemoglobin concentration) [8]	• Good discrimination power • Accurate outcome prediction when large sample size is utilized [5, 8] • Poor precision at the individual patient level [9] • AUC in previous studies: 80% [10], 83% [11], 85% [7] and 86% [8].
Corticosteroid Randomization After Significant Head injury (CRASH)	Adult patients (age ≥ 16 years) with TBI and GCS ≤14 (9)	Predicts the probabilities of 14-day mortality and 6-month unfavorable outcome [8]	Age, GCS, Pupils reactivity, major extracranial hemorrhage and CT findings (midline shift, obliteration of third ventricle, subarachnoid hemorrhage, petechial hemorrhage, and non-evacuated mass) [10]	• Good discrimination power [8] • Accurate outcome prediction when large sample size is utilized [8, 10] • Poor precision at the individual patient level [9] • AUC in previous studies: 86% [7], 87% [8] and 89% [10]
Marshall scale	Patients who sustained TBI	Grades the TBI and predicts the TBI outcomes on the basis of CT scan findings	Presence of mass lesion, midline shift, and status of the peri mesencephalic cisterns	• Simple to use • Reasonable discrimination power • Narrow scope (limited to 3 variables) • Limited applicability to clinical practice [12] • AUC in previous studies: 71% [13], 63.5% [14] and 78% [12]
Rotterdam CT scoring	Patients who sustained TBI	Grades the TBI and predicts the TBI outcomes on the basis of CT scan findings	Presence of mass lesion, midline shift, status of the peri mesencephalic cisterns and the presence of traumatic intra-ventricular or sub-arachnoid hemorrhage (tSAH) [13]	• Reasonable discrimination power • Does not differentiate between the type and size of the mass lesion [12] • AUC in previous studies: 69.8% [14] 84% [12] and 85% [15]
Helsinki Computerized Tomography Score Chart	Patients who sustained TBI	Grades the TBI and predicts the TBI outcomes on the basis of CT scan findings	Mass lesion type, Mass lesion size, presence of intraventricular hemorrhage, suprasellar cistern	• Superior to Marshall and Rotterdam scales • Good accuracy and discrimination power • Lower performance when used alone as a predictive method [12, 14] • Reported AUC: 71.7% [12] and 74.6% [14],

In addition, scholars designed several predictive models that aim to help the clinicians and the researchers to predict the TBI prognosis and outcomes. Jacobs et al. [16] designed a predictive model that aims to predict the outcomes of moderate to severe TBI using demographics, clinical data (e.g. vital signs, pupils reaction, and Glasgow coma scale (GCS)) and radiological parameters (Brain computed tomography scan (CT) findings). The study found that age, pupil responses, GCS score and the occurrence of a hypotensive episode post-injury and several CT scan findings are good predictors for the TBI outcomes.

The use of machine learning techniques to predict diseases outcomes has grown significantly in the last decade. Several studies proved that the machine learning predictive techniques outperformed the classical multivariate techniques [17, 18]. In a systematic review of 30 studies that used machine learning techniques to predict several neurosurgical outcomes including mortality following TBI, machine learning techniques outperformed several well-known classical predictive tools and performed similar or better than field experts in some instances [19]. Rau et al. [6] used machine learning techniques to predict the moderate to severe TBI mortality. The authors used age, sex, use of helmet, co-morbidities, GCS, and vital signs as predictors. They used Logistic Regression (LR), Artificial Neural Network (ANN), Decision Tree (DT), Support Vector Machine (SVM), and Naïve Bayes Classifier (NB) to classify the patients based on the survival outcomes. They compared the performance of the models in terms of accuracy, sensitivity, specificity and the area under the curve (AUC). ANN yielded the best performance amongst all with 96.8% AUC, 92% accuracy, 84.4% sensitivity and 92.8% specificity. Hale et al. [12] used machine learning technique (ANN) to predict 6- month favorable/unfavorable outcomes including mortality in 565 pediatric patients who sustained TBI. They used GCS, pupils reactivity to light, blood glucose level, blood hemoglobin concentration, mass lesion, traumatic sub-arachnoid hemorrhage (tSAH), cistern status, and midline shift to build the predictive model. Further, they compared the performance of the ANN based predictive models with three of the known classical predictive models, namely; Helsinki, Rotterdam, and Marshall. The machine learning model not only achieved profound accuracy (> 94%), but also outperformed the three classical predictive tools. This finding supports Eftekhar et al. [17] that found ANN to significantly outperform the logistic regression based predictive models in predicting diseases outcomes with AUC of 96.5% vs. 95.4%.

This study aims to design supervised machine learning predictive model to early predict in-hospital mortality in adult patients who sustained TBI and admitted to the level 1 trauma center of Hamad General Hospital (HGH)- Hamad Medical corporation (HMC); a governmental non-for-profit healthcare organization.

## Methodology

**Fig. 1:**
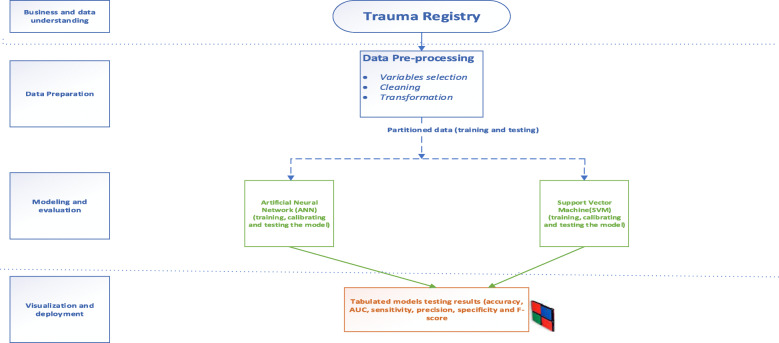
Research methodology

The study was conducted in accordance with the Cross-Industry Standard Process for Data Mining (CRISP-DM) that provides definition of typical phases of the data mining projects. CRISP-DM breaks data mining process into six phases: business and data understanding, data preparation, modeling, evaluation and deployment [20]. Figure 1 provides a summary of the methodology.

### Business and data understanding

Not all the registry data were usable in this study. Therefore, to better understand and choose meaningful variables, the authors explored the definition of each variable in the trauma registry data dictionary. In addition, authors reviewed the literature in order to determine which among the enormous number of variables need to be considered a predictor and which among them to be imputed if in case they have missing values [21]. Pediatric patients (< 14 years old) were excluded. This was important for understanding and interpreting the results as some of the important parameters (i.e. vital signs) are different between the pediatrics and adults groups.

### Data preparation

The study was approved by the Institutional Review Board (IRB MRC-01-19-106) of HMC. This retrospective study targeted all adult patients who were admitted to the trauma center at HGH in the period from January 2014 to February 2019 and registered in the trauma registry. A total of 2318 patients with TBI were registered in the trauma registry for the given period.

Only Adult patients (≥ 14-year-old) who sustained TBI were included in the study. All variables that have no predictive power (e.g. health record number, date of admission and date of disposition) or those that were severely imbalanced (e.g. gender: where female patients were less than 6%) were excluded. Missing data may seriously impact the predictive models performance [22]. Several approaches to handle data missingness were used in the literature such as elimination of the incomplete records [6] or imputing the missing values which is a widely used approach [22]. Due to the criticality of the subject, records with missing data were eliminated. Subsequently, 1620 eligible patients were included in the study.

The retrieved data included the following variables: Age, gender, mechanism of injury, mode of arrival, alcohol blood level, blood pressure, heart rate, Glasgow Coma Score (GCS), CT findings, intubation status and location, date/time of injury, time of admission to the Emergency Department (ED), patients known comorbidities, performed procedures, blood transfusion, administration of the Venous Thromboembolism (VTE) prophylaxis, blood transfusion, in-hospital complications, outcome and date of disposition.

Additional variables were secondarily generated from the retrieved variables: Shift of admission (D: 7 am to 6:59 pm and N: 7 pm to 6:59 am) [23, 24].

### Outcome measure

The outcome measure is the in-hospital mortality during the initial hospitalization post moderate to severe TBI. It is a dichotomous variable (0 = alive and 1 = dead). Patients who were discharged from the trauma surgery section or transferred to another hospital were considered alive.

### Prediction models

Two of the powerful supervised machine learning techniques were utilized to allow us to compare their performance with each other and with previous studies in order to recommend the model that achieves the optimal performance and highest practicality in supporting clinical decision. Artificial neural networks (ANN) and Support vector machines (SVM) are widely used in predicting in-hospital mortality. Therefore, they were selected to provide base line comparative performance. SPSS modeler 18.1 was used to conduct the analysis.

To prevent overfitting and to validate the models’ performance, we partitioned the data into training and testing sets and the overfit prevention was set at 30%. Table 2 explains the data partitions.

**Table 2:** data partitions

Set	Proportion	Number of cases	Number of alive patients	Number of dead patients
**Training set**	70%	1120	977	143
**Testing set**	30%	500	440	60
**Total**	**100%**	**1620**	**1417**	**203**

#### Artificial neural networks (ANN)

ANN are widely used machine learning technique that performs powerfully in classification and pattern identification [25]. When used for classification, ANN is seen as a set of connected input/output units in which each connection has a weight associated with it. This weight represents the strength of the connection between the units [26]. Scholars consider ANN as a black-box analytical model. Nonetheless, their great potentials in supporting clinical practice through the engagement with the evidence-based medicine are undeniable [12]. Usually, the performance of the neural network is optimized through partitioning the data into training and test data sets which helps preventing overfitting. The training continues until the error is no further reducible [27]. Once trained, the ANN can be used for future cases where the outcome is unknown [28].

#### Support vector machines (SVM)

SVM is a powerful classification machine learning algorithm that can be used for linear and non-linear data sets [25]. When using SVM for classification purpose, it is very important to decide which kernel function better achieves the optimal hyperplane that separates the classes [29]. Linear kernel was used in this study as it optimized the predictive performance in the preliminary assessment compared to other functions (i.e. polynomial, sigmoid or Radial Basis functions).

## Results

Among the 1620 patients who were included in this study, 203 (12.5%) died in the hospital during their initial hospitalization. Mean age was 34.4 years and mean age at death was 37.2 years. The most common mechanism of injury was fall from height (34%) followed by Motor vehicle crash (30%). The most common CT finding/mass lesion was subdural hemorrhage (28.1%) followed by extradural hemorrhage (22.9%) with 22% of the patients’ sample sustaining midline shift. Tables 3 and 4 show the sample characteristics and the descriptive statistics for the study sample.

**Table 3:** Sample characteristics- continuous variables

Variable	N	Mean	SD	Mean at death
**Age**	1620	34.4	13.9	37.2
**ED systolic blood pressure (SBP)**	1620	127.66	22.6	118
**ED heart rate (HR)**	1620	93	22.9	108.5

**Table 4:** Sample characteristics - Nominal and ordinal variables

Variable	Category	Count/%	With Outcome 0 (Alive)/%	With Outcome 1 (Dead)/%
**Race**	Asian	977/60.3%	858/87.8%	119/12.2%
	Other	643/39.7%	559/86.9	84/13.1%
	Total/%	1620/100%	1417/87.5%	203/12.5%
**Mechanism of Injury (MOI)**	MVC^1^	486/30%	413/85%	73/15%
	Fall	551/34%	495/89.8%	56/10.2%
	Pedestrian	268/16.5%	216/80.6%	52/19.4%
	Other	315/19.4%	293/93%	22/7%
	Total/%	1620/100%	1417/87.5%	203/12.5%
**Arrival mode**	Ambulance	1350/83.3%	1167/86.4%	183/13.5%
	Other	270/16.7%	250/92.6%	20/7.4%
	Total/%	1620/100%	1417/87.5%	203/12.5%
**Midline shift**	No	1260/77.8%	1155/91.7%	105/8.3%
	Yes	360/22.2%	262/72.8%	98/27.2%
	Total/%	1620/100%	1417/87.5%	203/12.5%
**CT findings/mass lesion**	SDH^2^	455/28.1%	380/83.5%	75/16.5%
	EDH^3^	371/22.9%	352/94.9%	19/5.1%
	SAH^4^	152/9.4%	114/75%	38/25%
	CONT^5^	321/19.8%	303/94.4%	18/5.6%
	DAI^6^	120/7.4%	99/82.5%	21/17.5%
	Other	201/12.4%	169/84.1%	32/15.9%
	Total/%	1620/100%	1417/87.5%	203/12.5%
**Cerebral edema**	No	1517/93.6%	1370/90.3%	147/9.7%
	Yes	103/6.4%	47/45.6%	56/54.4%
	Total/%	1620/100%	1417/87.5%	203/12.5%
**Facial bones fracture**	No	981/60.6%	857/87.4%	124/12.6%
	Yes	639/39.4	560/87.6%	79/12.4
	Total/%	1620/100%	1417/87.5%	203/12.5%
**Lung contusion**	No	1273/78.6%	1152/90.5%	121/9.5%
	Yes	347/21.4%	265/76.4%	82/23.6%
	Total/%	1620/100%	1417/87.5%	203/12.5%
**Hemothorax**	No	1482/91.5%	1319/89%	163/11%
	Yes	138/8.5%	98/71%	40/29%
	Total/%	1620/100%	1417/87.5%	203/12.5%
**Pneumothorax**	No	1387/85.6%	1251/90.2%	136/9.8%
	Yes	233/14.4%	166/71.2%	67/28.8%
	Total/%	1620/100%	1417/87.5%	203/12.5%
**Abdominal organ injuries**	No	1417/87.5%	1278/90.2%	139/9.8%
	Yes	203/12.5%	139/68.5%	64/31.5%
	Total/%	1620/100%	1417/87.5%	203/12.5%
**GCS category**	13–15	893/55.1%	875/98%	18/2%
	9–12	122/7.5%	113/92.6%	9/4.4%
	≤ 8	605/37.3%	429/70.9%	176/29.1%
	Total/%	1620/100%	1417/87.5%	203/12.5%
**Shift**	7 am-6:59 pm	858/53%	758/88.3%	100/11.7%
	7 pm-6:59 pm	762/47%	659/86.5%	103/13.5%
	Total/%	1620/100%	1417/87.5%	203/12.5%
**Known comorbidities**	No	1328/82%	1167/87.9%	161/12.1%
	Yes	292/18%	250/85.6%	42/14.4%
	Total/%	1620/100%	1417/87.5%	203/12.5%
**Intubation**	No	848/52.3%	847/99.9%	1/0.1%
	Yes	772/47.7%	570/73.8%	202/26.2%
	Total/%	1620/100%	1417/87.5%	203/12.5%
**VTE**^**7**^**prophylaxis**	No	656/40.5%	537/81.9%	119/18.1%
	Yes	964/59.5%	880/91.3%	84/8.7%
	Total/%	1620/100%	1417/87.5%	203/12.5%
**Blood transfusion**	No	1013/62.5%	989/97.6%	24/2.4%
	Yes	607/37.5%	428/70.5%	179/29.5%
	Total/%	1620/100%	1417/87.5%	203/12.5%

(1) *MVC* Motor Vehicle Crash, (2) *SDH* Subdural Hemorrhage, (3) *EDH* Epidural Hemorrhage, (4) *SAH* Subarachnoid Hemorrhage, (5) *CONT* Hemorrhagic Contusion, (6) *DAI* Diffuse Axonal Injury, (7) *VTE* Venous Thromboembolism

### Performance of the data mining techniques

To calculate the models’ performance metrics, we first constructed the confusion matrix that displays the relationship between the actual observations and the predicted conditions.

Table 5 shows the performance evaluation metrics for the two machine learning techniques in the test data partition. Both models achieved accuracy greater than 91%. Nevertheless, since accuracy alone is insufficient measure to evaluate model performance, AUC, precision, NPV, sensitivity, specificity and F-score measures were taken into consideration. SVM achieved the best performance (Table 5).

**Table 5:** Performance of the classification models

Model	Number of predictors	Accuracy (%)	AUC (%)	PPV (%)	NPV (%)	Sensitivity (%)	Specificity (%)	F-Score
**SVM**	21	95.6	96	88	97	73	99	0.8
**ANN**	21	91.6	93.5	66	96	62	96	0.64

### In-hospital mortality risk factors

**Fig. 2:**
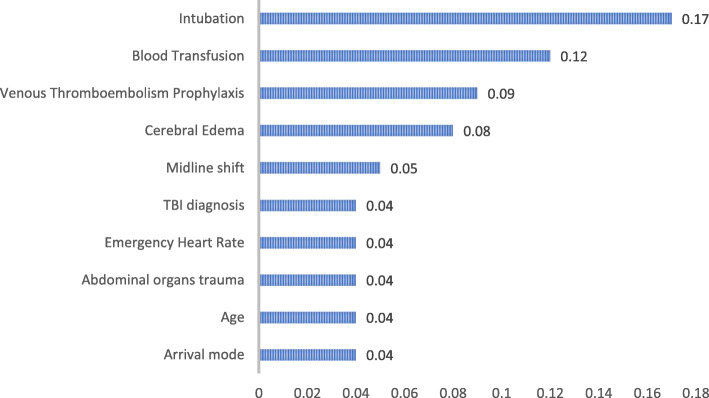
Importance of predictors in Support Vector Machines

SVM utilized all the 21 variables in predicting the in-hospital mortality. In machine learning, the contribution of every predictor to the overall model’s capacity to produce accurate predictions is usually presented in the form of predictor’s importance (Fig. 2) [30]. The first predictor is usually the most important predictor to the model’s capacity. Then the other predictors importance values are ranked in relation to the first ranked predictor. SVM revealed that receiving endotracheal intubation during resuscitation plays the most important role in predicting the in-hospital mortality.

## Discussion

The early prediction of in-hospital mortality in patients with traumatic brain injury is of utmost importance. Early and powerful prediction of mortality helps clinicians and healthcare managers to optimize the management of medical resources, initiate appropriate diagnostics and interventions in a timely manner, conduct comparative audits and ensure that the patients’ families and others receive appropriate guidance [3, 6]. However, prediction of disease prognosis and outcomes requires developing good prognostic models that include large samples and enjoy high external and internal validity in order to be generalizable beyond a specific research setting [31]. Many prognostic models were published over the years but only few of them achieved sample validity requirements [32]. Usually, clinicians use certain prognostic factors such as GCS to guide their therapeutic decisions and to estimate prognostic outcomes [3, 32]. Nonetheless, such predictors may be affected by several factors such as alcohol use which negatively affects the prediction success and the discrimination power of the model [11, 12]. Thus, for accurate outcome prediction, multiple risk factors (e.g. age, GCS and others) need to be considered jointly in developing prognostic model [32, 33].

In terms of models’ performance, SVM outperformed the ANN in all the performance evaluation metrics (Table 5). Therefore, SVM is the chosen model for deployment.

At a wider scale, in this study, the SVM outperformed the conventional multivariate LR based models that utilize the conventional TBI prognostic models as reported in Table 1. The highest reported AUC when using the conventional prognostic models was 92% [10, 12, 16]. Furthermore, when comparing this study’s machine learning models’ performance with the published literature on TBI, we found that the performance of the SVM model was higher or similar to the performance of the machine learning models in similar studies [6, 19]. This comparison is crucial when the external validity of this study was considered.

This study ranked the intubation to be the most important predictor for post TBI in-hospital mortality. Almost 26% of patients who were intubated in the first 24 h post injury died during their initial hospitalization compared to 0.1% of those who were not intubated. This could be attributed to the severity of TBI as the severer the injury the higher likelihood to get intubated. Moreover, intubation increases the length of stay in the hospital and increases the risks of in-hospital complications e.g. ventilator associated pneumonia that contributes significantly to increasing the mortality [34]. The need for blood transfusion during resuscitation has a significant relationship with the in-hospital mortality. 29% of patients who received blood transfusion during resuscitation died compared to 2.4% mortality among those who didn’t received blood. The need and the consequences of blood transfusion in TBI are still debatable. Several studies reported that blood transfusion in TBI is associated with unfavorable outcomes [35, 36]. This could also be explained by the fact that patients who needed blood transfusion were those who had severer injuries and had lost significant amounts of blood. Therefore, these patients are prone to poor TBI outcomes already.

Consistent with the previous literature, this study found that patients who received venous thromboembolism (VTE) prophylaxis had better survival rate compared to those who didn’t [37]. Almost 18% of those who didn’t receive the VTE prophylaxis deceased during their initial hospitalization compared to 8.7% of those who received VTE prophylaxis. Also, this study found that 54.4%of patients who developed cerebral edema following the primary TBI have died in-hospital compared 9.7% of those who didn’t develop cerebral edema. This finding is consistent with Jha et al. who reported that cerebral edema is a leading cause of in-hospital mortality as it occurs in more than 60% of patients with mass lesions including post TBI hemorrhage [38]. Cerebral edema is a secondary complication to the TBI when the brain tissue water increases following the injury. Hence, significant efforts in TBI management go to the prevention of the secondary brain injury and to maintain adequate cerebral perfusion pressure (CPP) [39, 40]. Midline shift is a major post traumatic complication that leads to serious unfavorable effects including mortality [8, 12, 41]. Around 27% of patients who had midline shift deceased compared to 8.3% of those who had no midline shift reported in their CT scan. TBI diagnosis as per brain CT scan result plays an integral role in predicting in-hospital mortality post TBI. Interestingly, 25% of those who had subarachnoid hemorrhage (SAH) following the TBI died compared to 17.5% and 16.5% for those with DAI and SDH respectively. It is documented in the literature that traumatic SAH has a significant effect on the in-hospital mortality [8, 12, 19]. Further, presenting heart rate (HR) is an indicator of the patient’s hemodynamic stability following any type of trauma particularly TBI. High HR (> 100 bpm) [42] especially when associated with Low SBP (< 90 mmHg) [40] may indicate hypovolemic shock state which leads to poor CPP. This study found a positive relationship between the HR and in-hospital mortality. The HR in this study was collected upon arrival to the ED following trauma. The mean HR upon admission was 93 bpm. The mean HR for those who survived was 90.8 while it was 108.5 bpm for those who later died in the hospital. Interestingly, the mortality rate increases significantly when patients with TBI have associated abdominal injuries [43]. Mortality among those with associated abdominal injury is 31.5% compared to 9.8% among those with no associated abdominal injury.

Finally, the 10th ranked important variable was the arrival mode. Patients that arrived to the trauma center via ambulance had higher mortality compared to those who arrived to the trauma center via another mode (13.5% vs. 7.4%). This is consistent with previous literature which found that the mortality patterns are affected by the mode and the time of arrival to the emergency room following TBI or polytrauma [44, 45]. This could be due to the assumption that the time between injury and arrival of the ambulance then the arrival to the hospital is relatively longer than arrival with private vehicle [45] or simply by the assumption that the severer the injury the higher the likelihood that a patient gets transported to the hospital via ambulance.

### Limitations

One of the most important limitations in this study was faced during data processing and preparation. Several variables in the registry are recorded as text-free which complicates data preparation process. Data were abstracted from Qatar national trauma registry; which is contributing data to the National Trauma Data Bank (NTDB) and the Trauma Quality Improvement Program (TQIP) of the American College of Surgeons-Committee on Trauma (ACS-COT). Therefore, there are several potentially useful predictors that were unobtainable such as laboratory results and received medication. The deployment of the model to support clinical decision making is another significant challenge. This is due to several reasons such as the questionable reliability of the non-traditional predictive techniques that stems to a certain extent from the lack of awareness among the clinicians about the artificial intelligence potentials in supporting clinical decision-making process. Very importantly, unlike the logistic regression for instance, the standardized coefficients and the odds ratios pertaining to each predictor in the SVM are not obtainable. This makes the results interpretation more complex than the traditional computational techniques.

## Conclusions

This study demonstrated that the performance of the machine learning techniques is superior to the conventional multivariate models. Furthermore, the results were consistent with the known body of knowledge. Thus, with the availability of massive data sets in the electronic medical records and other structured registries, clinical evidence could be made available quickly and with less effort.

From another perspective, the results of this study may encourage the decision makers in the trauma surgery to integrate the machine learning techniques with the trauma registry and the electronic medical records. This may help clinicians plan their preventive efforts and mobilize the necessary resources in an early stage of patient treatment which could improve the care outcomes.

## Data Availability

n/a
